# Exploring the User Interaction Network in an Anxiety Disorder Online Community: An Exponential Random Graph Model with Topical and Emotional Effects

**DOI:** 10.3390/ijerph19116354

**Published:** 2022-05-24

**Authors:** Jingfang Liu, Yafei Liu

**Affiliations:** School of Management, Shanghai University, 99 Shangda Road, Shanghai 201900, China; jingfangliu@shu.edu.cn

**Keywords:** online health community, anxiety disorder, exponential random graph model, interactive network, topic effect, emotional effect

## Abstract

The increasing number of people with anxiety disorders presents challenges when gathering health information. Users in anxiety disorder online communities (ADOCs) share and obtain a variety of health information, such as treatment experience, drug efficacy, and emotional support. This interaction alleviates the difficulties involved in obtaining health information. Users engage in community interaction via posts, comments, and replies, which promotes the development of an online community as well as the wellbeing of community users, and research concerning the formation mechanism of the user interaction network in ADOCs could be beneficial to users. Taking the Anxiety Disorder Post Bar as the research object, this study constructed an ADOC user interaction network based on users’ posts, comments, and personal information data. With the help of exponential random graph models (ERGMs), we studied the effects of the network structure, user attributes, topics, and emotional intensity on user interaction networks. We found that there was significant reciprocity in the user interaction network in ADOCs. In terms of user attributes, gender homogeneity had no impact on the formation of the user interaction network. Experienced users in the community had obvious advantages, and experienced users could obtain replies more easily from other members. In terms of topics, pathology popularization showed obvious homogeneity, and symptoms of generalized anxiety disorder showed obvious heterogeneity. In terms of emotional intensity, users with polarized emotions were more likely to receive replies from users with positive emotions. The probability of interaction between two users with negative emotions was small, and users with opposite emotional polarity tended to interact, especially when the interaction was initiated by users with positive emotions.

## 1. Introduction

With the rapid development of the economy and the improvement of living standards, people have been growing more concerned about not only their physical health but also their mental health. Correspondingly, the need for health information such as treatment experiences, drug efficacy, and emotional support has also been increasing. Traditional face-to-face communication has not been able to meet the growing mental health demands. The emergence of online health communities has provided an effective means for the rational allocation of medical resources [1]. Medical service personnel can use their leisure time to provide patients with online consultation services [1]. Similarly, people seek health information online and communicate health statuses with others without the limitations of time or location [2].

Anxiety disorder is a psychological disorder requiring long-term treatment. Due to social prejudice and discrimination [3], people with anxiety disorder are often unwilling to communicate their disorder status face-to-face. The emergence of anxiety disorder online communities (ADOCs) organized by relevant professionals has provided patients with an online channel to seek health information and communicate health statuses.

Users in an ADOC discuss different topics through a series of content creation behaviors such as posting, commenting, and forwarding. The interactive network between users in a community is also formed in the user communication process. An ADOC is different from a traditional entertainment online community. Users in an ADOC often have clear health information needs [2] and high emotional intensity [4]. Patients receive emotional support from communication with others. It is one of the main benefits of participating in an online health community. In addition, users participate in different topical discussions according to their own needs and preferences. As a result, a user interaction network in an ADOC is formed. At present, there are often a large number of posts without replies in ADOCs. This hinders the communication and interaction between users and discourages the participation of newly registered users in the community. Newly registered users are typically inexperienced. With the continuous entry of inexperienced users, it is important to study how the topics and the emotional intensity of user created content affects the user interaction network. The purpose of this study was to explore the communication mode of users in ADOCs and analyze the influences on the network structure, user attributes, topics, and emotions on the formation of a user interaction network. First, studying the formation mechanism of user interaction networks in ADOCs promotes the interaction between users in the community. It has also been shown to enhance the community participation and to encourage inexperienced users to take part [5]. Second, it supports community managers as they consider corresponding measures to improve user activity and promote the success and construction of the community.

## 2. Literature Review

### 2.1. Research into Online Health Communities

Online communities are platforms for communication and interaction using the Internet. They are widely used in the education and entertainment industries, and they have developed significantly in the field of medical and health care, such as WebMD, MedHelp, Yahoo Health, 39.net, haodf.com, and chunyuyisheng.com. These online health communities (OHCs), organized and managed by relevant professionals in the industry, have not only enabled medical professionals to provide remote online consulting services, but they have also allowed laypeople to exchange treatment experiences and provide emotional support. The vigorous development of online health communities has provided a new service model for the medical industry. With an increasing number of users in online health community, research into these communities has become a focus. Thus far, the research into online health communities has included aspects such as the community itself, users, and information exchange. The community dimension has considered the operating modes of patient interaction, doctor–patient interaction, and doctor interaction in a community. The user dimension has focused on the overall structure of the user relationship network and the motivation and influencing factors of user behaviors. The information dimension has explored the topics of user-generated content and information privacy. In terms of the community dimension, Guo et al. studied the social and economic advantages doctors received by participating in an online health community from the two dimensions of doctors’ status and professional knowledge, and then they discussed their impacts on different groups of doctors. The study found that in addition to a doctor’s status and reputation, the professional level of doctors was an important part of determining their returns in OHCs [6]. In terms of the user dimension, Li et al. studied the network density, centrality, and other network structure characteristics of the information exchange network of traditional Chinese medicine students in the Clove Forum, a large-scale medical professional exchange forum in China, and its impact on their social capital [7]. Atanasova et al. found that the interaction among patients in an online health community provided users with health information and emotional support [8]. Yan et al. studied the influential factors of members’ knowledge-sharing in an online health community based on social exchange theory. The study found that self-worth, members’ sense of social support, reputation improvement, initial concerns, execution costs, and cognitive costs had different degrees of impact on members’ knowledge-sharing [9]. Zhang et al. identified the importance of reciprocity in member interactions in online health communities [10]. In terms of the information dimension, Kisekka et al. explored the relationship between information security and cancer patients’ attitudes toward exchanging health information, the frequency of access to health records, and the perception of nursing quality from three aspects: beliefs concerning information security, privacy, and trust in health information. The study found that an increase in privacy issues reduced the frequency of patients’ use of health records and reduced their positive attitudes toward exchanging health information and their views on the quality of care [11]. Lederman et al. developed a new model for the trustworthiness of information in online health forums. The study found that different assessment criteria were used according to the needs of different participants [12]. The current research into online health communities was less concerned with user relationship networks; instead, the objective of this study was on user relationship networks and the overall network structure to explore of the formation mechanism behind the network structure. Therefore, it was necessary to study the formation mechanism of user relationship networks in online health communities.

### 2.2. Research into Online Communities from the Network Perspective

In an online community, users form a relationship network by engaging and showing concern via posting and responding to others. At present, many researchers have studied online communities from the perspective of networks, and the research method has been focused on social network analysis. The types of online communities studied in the existing literature have included social, academic, and question-and-answer (Q&A) online communities. Shiau et al. found the network structure of the clustering among papers through a social network analysis of papers published on academic websites [13]. Sun studied the conversation network structure of Twitter chat communities with the help of an exponential random graph model and found that the user conversation process was composed of individual, binary, and ternary levels [14]. Said et al. conducted social network analysis on the text information published by users in the Twitter community and tested the small-world characteristics of Twitter [15]. Saqr et al. selected the discussion data of a teaching case in an online learning community and analyzed the network structure of the user interaction network in the online learning community from the perspective of social capital, and they found that the role, position, and social capital of students in the online community could impact the network structure [16]. Xu et al. analyzed the posts of users in an online community and found that there was obvious homogeneity in the user interaction network. Two users who used tags were more likely to establish contact, while users who did not use tags were less likely to contact each other [17].

Through the literature review, we found that the existing research into online health communities mostly focused on users, and research on user relationship networks was relatively scarce. Studying online communities from the perspective of networks has been a current focus. However, most of the existing studies have focused on social online communities, and few scholars have studied online health communities, especially those focused on mental disorders, from the network perspective. The ADOC itself is an online health community for psychological disorders. Users participate in different topics according to their interests and needs. Furthermore, users’ posts and comments in the community contain a large number of emotional expressions. Therefore, based on the user data of the Anxiety Disorder Post Bar and with the help of an exponential random graph model, this study explored the formation mechanism of the user interaction network and the effects of the network structure, user attributes, topics, and emotional intensity. This study supports communication between users in ADOCs and may improve the activity of experienced users and the longevity of new/inexperienced users, which could improve the success of these communities.

## 3. Theoretical Basis and Research Assumptions

An ADOC is an online mental health community that provides communication platforms and information on anxiety disorders. The various content creation behaviors of users in ADOCs, such as posting, comments, and replies, and the interaction between these users form a user interaction network. To explore how the network structure and user attributes affected the formation of relationships within the network, this study proposed a series of hypotheses and empirical research based on community characteristics and previous theories.

Reciprocity refers to the characteristic that two nodes tend to connect with each other in a directed network [18]. Reciprocity is a common relationship in human society and is often used to study human social networks. According to social exchange theory, while providing valuable resources to others, individuals also expect to be rewarded by each other [19]. These resources can be economic, social, or various other resources. For example, tangible economic resources such as information, consultation, and services and intangible social resources such as friendship and reputation have been used as social exchange resources [20]. In previous studies, many researchers have incorporated reciprocity into social networks [21,22,23]. Research has shown that reciprocity had an important structural effect on social networks, also known as an endogenous effect [24]. In an ADOC, some users obtained replies from other members via posts seeking help. After receiving a reply, there may be further communication from both sides, which reflects the reciprocal relationship in the social network. Therefore, we speculated that reciprocity could promote the formation of user relationships in ADOCs and proposed the following hypothesis:

**H1:**  *Users tend to have reciprocal interactions in ADOCs.*

A social network is formed by the interconnection of many nodes in a network. Therefore, the formation of network relationships is related not only to network self-organization but also to the attributes of the nodes in the network, which play an important role in the formation of network relationships [25]. According to the principle of homogeneity, when two nodes in a network have similar attributes, they are more likely to establish contact [26]. If users in ADOCs consider the other party to be similar to themselves in some attributes when commenting or replying, homogeneity will arise. When studying homogeneity, demographic characteristics, such as race, age, gender, and living area, have often been analyzed as important factors affecting the formation of social networks [27,28,29]. Sun studied the formation process of conversation network structures in Twitter chat communities, used user identity and account types as user attributes, and then discussed the impact of homogeneity on the formation of conversation networks in Twitter communities [14]. Song et al. applied homogeneity theory to an online medical community and studied the impact of gender, treatment, and health status homogeneity on the formation of patient friendship networks in the community [27]. In an online community, users have different needs and interests. According to these needs and preferences, users will participate in different topic discussions. In the existing research, few researchers have explored the role of topic homogeneity in the formation of social networks. According to previous studies and the characteristics of ADOCs, we explored whether gender and topic preference homogeneity would affect the formation of user interaction networks in ADOCs. Therefore, this paper proposed the following hypotheses:

**H2:**  *Users of the same gender are more likely to interact in ADOCs.*

**H3:**  *Users who prefer the same topic are more likely to interact in ADOCs.*

According to the priority connection principle in social networks, the newly added nodes in the network would tend to establish connections with the nodes with higher network centrality among the existing nodes [30]. The length of time users participated in a community reflected their social capital in the community to a certain extent. Experienced users participate in a community by posting, replying, and commenting in the community and have established relationships with other members of the community. The relationships themselves have been identified as a type of social capital [31]. According to social capital theory, users with higher social capital had the advantage of obtaining resources in a social network [32]. Xiong et al. studied an online government community’s user interaction network and found that users with higher social influence were more likely to obtain replies from others [33]. In addition, the research of Goette et al. also indicated that the formation of social networks was closely related to users’ personal information [34]. Therefore, we predicted that experienced users would be more likely to receive replies from other users after posting or commenting in a community. In addition, we expected that it would likely be experienced users who provided social support in an online community for anxiety. Some experienced users would support inexperienced users who joined the community, inexperienced users would express their gratitude to them after receiving support, and the interactive relationships would be established as a result of this process. Therefore, this paper proposed the following hypothesis:

**H4:**  *Experienced users are more likely to receive replies from other users in ADOCs.*

As one of the important factors affecting social media [33], emotion was likely to have an important impact on user interaction networks. According to the theory of emotional contagion, the emotional state of an individual or group could affect the emotional state of another individual or group [35]. Information expressing polarized emotions was more likely to concern others and then spread among individuals or groups. Fan et al. found that users’ emotional intensity could affect the dissemination of information on social media [36]. In addition, Brady et al. found that polarized emotions affected users’ participation in social networks [35]. In an online community, users’ emotions are spread through posts, comments, replies, and other content. Therefore, written expressions in an online community can contain all types of user emotions. An ADOC is an online mental health community, and its nature also determined that users in the community were more likely to express positive or negative emotions in posts, comments, and replies. These emotions concerned other users, who would then reply. In previous studies, researchers have studied the impact of emotional expression on user response networks [33]. However, it is not yet clear whether polarized emotions have been more likely to receive positive or negative responses. On the one hand, according to homogeneity theory, we expected that users expressing the same emotional polarity were more likely to interact. On the other hand, we observed that in the ADOC, users received emotional support from other users after posting or commenting with negative emotions, and these responses generally included positive emotions. Therefore, based on this, we proposed the following hypothesis:

**H5:**  *Users with polarized emotions are more likely to receive replies from users with positive emotions in ADOCs.*

## 4. Materials and Methods

### 4.1. Data Sources

The data were sourced from the Anxiety Disorder Post Bar. The Anxiety Disorder Post Bar is one of the communities with the most users among the online communities. The users in the community were predominantly receiving treatment for their disorder. They could communicate, seek help, share disorder knowledge, and engage in other actions in the community. By 2021, the number of posts had reached 3.586 million. This study obtained the posting and comment data of users and the attribute data of the relevant users in the Anxiety Disorder Post Bar. To ensure the integrity of user attributes, we screened the data and finally obtained the complete attribute information of 6158 users. The user communication network formed by these users contained 16,521 edges.

### 4.2. Data Processing

Users participated in discussions on different topics in the community and expressed different emotional intensities according to the text data. To explore whether these different topic preferences and emotional intensities affected the interaction between users and how they affected the communication and interaction between users, in this study, the user post and comment data obtained by text mining were further processed. Seven different topics, including social support (SS), pathology popularization (PP), drug efficacy (DE), emotional catharsis (EC), panic attack records (PAR), symptoms of generalized anxiety disorder (SGAD), and diagnosis and examination of hypochondriasis (DEH), were obtained. Social support referred to sharing treatment experiences and providing emotional support. Drug efficacy referred to the effects and side effects of taking anti-anxiety medications. Symptoms of generalized anxiety disorder referred to the symptoms related to generalized anxiety disorder. Emotional catharsis referred to users expressing negative emotions. Panic attack records referred to the experience and symptoms of a panic attack. Diagnosis and examination of hypochondriasis referred to suspected anxiety and going to the hospital for examination. Pathology popularization referred to popular science and explanations related to anxiety. In addition, we calculated the emotional score of each user’s text data through emotional analysis and obtained the average of each user’s emotional score.

The measurement methods of user node attributes are provided in Table 1. Gender was a binary classification variable with a value of 1 for males and 0 for females. The user registration time was a binary classification variable. According to the user registration time, we divided the users in the community into experienced and inexperienced users, attributed the newly registered users (25% of the total users) as inexperienced users and the other users as experienced users, and set the experienced users as 1 and the inexperienced users as 0. To obtain the emotional score of each text, we averaged the emotional score of all texts for each user to obtain their emotional polarity. Affective polarity was a categorical variable. The value of positive emotion was 2, the value of negative emotion was 1, and the value of neutral emotion was 0 [25]. This study analyzed the network effect of emotional polarity on user interactions. Therefore, the top 10% of users with the highest emotional scores were regarded as users with a positive emotional tendency, the 10% of users with the lowest emotional scores were regarded as users with a negative emotional tendency, and the other users were regarded as users with neutral emotions [25]. The topic type was a classified variable. Through topic analysis, we classified the posts and comments published by users of the ADOC. They were divided into 7 topic types: 1 for pathology popularization, 2 for symptoms of generalized anxiety disorder, 3 for panic attack records, 4 for emotional catharsis, 5 for drug efficacy, 6 for diagnosis and examination of hypochondriasis, and 7 for social support. According to the frequency of the different types of topics discussed by users, the topic with the highest frequency discussed by users was regarded as a preferred topic and assigned a value.

### 4.3. Exponential Random Graph Model

This study used an exponential random graph model (ERGM) to test the proposed hypotheses. The ERGM is a statistical model based on relational patterns that can handle complex dependencies in a network [37]. The exponential random graph model has been widely used for social network analysis. It could not only explain the relationship patterns between individuals in a network but also explained the reasons for the formation of these relationships [38]. The parameter estimation results of the model corresponded to the probability of the network structure appearing in the network.

In the network diagram, n represents the number of nodes, and the relationship between nodes is represented by a random variable y_ij_. If y_ij_ = 1, it indicates that there is a connection between node i and node j; otherwise, there is no connection between node i and node j. The general expression of the ERGM is

(1) $$(P\left(Y=y|\theta \right)=\left(\frac{1}{c}\right)\exp\sum_{k=1}\theta_{k}^{T}z_{k}\left(y\right)$$

where Y represents the complete set of adjacency matrices of random variables, y represents the adjacency matrix of a certain random variable, and P(Y=y|θ) represents the probability of a certain network structure y in the complete set of network relations Y under the conditions. The variable c is a standardized constant to ensure that the probability of the model was distributed between 0 and 1. The variable k represents a certain network structure relationship; $\theta_{k}^{T}$ represents the estimated value of the parameter of the network structure k. If the estimated value of the parameter is positive, it means that there is a tendency for the structure to appear in the network, and the probability of the structure appearing in the network is higher than that of a random network. Conversely, the probability of the structure appearing in the network is lower than its probability of appearing in a random network. The variable $z_{k}\left(y\right)$ represents the statistic corresponding to the network structure y.

This article synthesized the characteristics of users and their posts and comments in the ADOC and other previous studies on similar networks and related theories and selected user gender, user registration time, the emotional polarity of the content posted by users, and preferred topic types as node attribute variables. The variables and measurement indicators are shown in Table 1, and the network structure diagram corresponding to each hypothesis is shown in Table 2. The ERGM was used to estimate whether and how these attributes affected the formation of user interaction networks in ADOCs.

## 5. Results and Discussion

### 5.1. ERGM Test Results

The test results of the exponential random graph model are shown in Table 3 as along with the corresponding parameter estimates and *p* values. According to existing research, the network structure corresponding to the positive parameter estimate is easier to form than the random network structure. That is, if the parameter estimate of the model is positive and significant, then the probability of occurrence in the network structure corresponding to the parameter estimation is higher than that of its occurrence in the random network structure [37].

#### 5.1.1. Network Structure Effects

In terms of network structure effects, Table 3 shows that the estimated value of parameter H1 was 2.898, and the *p* value was significant, indicating that the probability of reciprocity appearing in the ADOC was greater. Therefore, H1 was verified. Users in the ADOC tended to exchange information with each other. In the Anxiety Disorder Post Bar, we observed that some users received help from other users in the community after posting for help. Users who received help would also express their gratitude through posted replies. In addition, users in the community exchanged information regarding anxiety with each other in the form of posting replies. These processes also embodied the principle of reciprocity. Some studies have also found that reciprocal motivation played a significant role in users’ willingness to share knowledge in online health communities [10], which explained why there was a significant network structure of reciprocity in anxiety disorder online communities. Reciprocity promoted communication among users in ADOCs and enhanced community activity. It was also an important foundation for the development of the community and the wellbeing of community users.

#### 5.1.2. User Attribute Effects

In terms of user attribute effects, Table 3 shows that the estimated values of the gender parameters were negative and nonsignificant, which indicated that same-sex interactions had little influence on the formation of the network structures in ADOCs. The reason for this result could have been that opposite genders were attracted to each other, which led to interactions between the same genders having no significant influence on the interaction of users in ADOCs. This result was consistent with previous research [33]. In short, H2 was not verified.

The parameter estimation value of the user registration time was 0.103, and the *p* value was significant, indicating that the experienced users who had been registered for a long time had a higher probability of receiving replies from others in ADOCs. In short, H4 was verified. Experienced users were usually accompanied by a higher user level. Users could increase their personal level by signing in, posting, and replying to posts. High-level experienced users posted a large number of posts in the community or frequently replied to other users. These experienced users’ posts received more attention from other members of the community. At the same time, some studies also showed that trust had an important impact on the interaction between users [20]. Experienced users have participated in the community for a long time and established long-term connections with other members of the community, increasing the credibility of their shared information. Therefore, there were more replies under the experienced users’ posts in the Anxiety Disorder Post Bar. To some extent, it could also explain why experienced users received more responses in the community.

Experienced users played a decisive role in the development of the ADOC. We observed that some inexperienced users in the Anxiety Disorder Post Bar usually posted for help, and most of the responses they received were from experienced users. Improving the activeness of experienced users in the community could not only promote the interaction between inexperienced users and experienced users but also enhanced their sense of belonging among inexperienced users in the community, thereby promoting the sustainable development of the community and the wellbeing of community users.

#### 5.1.3. Topic Effects of User Texts

The topics of user texts, according to Table 3, were “topic_type_PP”, “topic_type_SGAD”, “topic_type_PAR”, “topic_type_EC”, “topic_type_DE”, “topic_type_DEH”, and “topic_type_SS”, respectively, represented pathology popularization (PP), symptoms of generalized anxiety disorder (SGAD), panic attack records (PAR), emotional catharsis (EC), drug efficacy (DE), diagnosis and examination of hypochondriasis (DEH), and social support (SS). Table 3 shows that the effects of pathology popularization were positive and significant. This showed that there was a greater probability of interaction between users who preferred pathology popularization. This also showed that users who prefer pathology popularization were more likely to gather together to discuss topics. The community can increase the section of pathology popularization. First, it provided disorder information for patients in ADOCs. Second, it was conducive to the subdivision management of the community content. In addition, we found that the topic effect of symptoms of generalized anxiety disorder in Table 3 was negative and significant. This indicated that users who preferred to discuss disorder symptoms did not specifically discuss the topic; the reason for this could be that users discussed other topics, such as emotional catharsis and drug efficacy, while discussing symptoms of anxiety disorders. In Table 3, we also found that other topic types had no significant impact on whether there was an interaction between users. The reason for this may be that these users participated in various topics in the community, which also led to their interaction with users with different topic preferences. In short, part of hypothesis H3 was supported. The results also indicated that some users would interact due to different topic preferences, which provided scientific guidance for the construction and development of ADOCs. The builders of the community can divide the community into different sections, which helps users obtain information more conveniently and at the same time can promote communication between people who preferred different topics.

#### 5.1.4. Emotional Effect of User Texts

Regarding the emotional impact of user text data, the second column in Table 3 is the parameter name of the relevant attribute. Among them, “positive_positive” represented a directed vector from positive emotions to positive emotions in the network, in other words, the responses of users with positive emotions to users with positive emotions. In the same way, “negative_negative” represented the response from users with negative emotions to users with negative emotions. “Positive_negative” represented the response from users with positive emotions to users with negative emotions. “Negative_positive” represented the response from users with negative emotions to users with positive emotions. Table 3 shows that the estimated value of the parameter of the same positive emotion was 0.413, and the *p* value was significant, indicating that the probability of interaction between two users with the same positive emotion was greater. The estimated value of the parameter of the same negative emotion was negative, and the *p* value was significant, indicating that the probability of interaction between two users with the same negative emotion was small. In the Anxiety Disorder Post Bar, users who posted negative messages hoped to receive support in the form of shared emotions or information, and supportive responses were typically not negative texts, which could also explain why there was little interaction between users with negative emotions. In addition, the results in Table 3 showed that the parameter estimates of the “positive_negative” attribute were positive, and the *p* value was significant; furthermore, the parameter estimates of the “negative_positive” attribute were negative, and the *p* value was significant. These two results indicated that the negative emotions of users were more likely to obtain replies from users with positive emotions, and users with positive emotions were more likely to obtain replies from users without negative emotions. In previous studies, some researchers have considered the emotional effects of user messages [25], but the results of this study were different. The communities studied were different. The communities previously studied were online health communities for physiological disorders. In addition, the samples used were also different. In short, H5 was supported by our results. These results also indicated that users with positive emotions played an important role in promoting communication among users in ADOCs. Users with positive emotions could provide emotional or informational support to users with negative emotions by responding to them, which also helped users with negative emotions by alleviating their negative emotions.

### 5.2. Robustness Test Results

This research used five models to test the robustness of the basic model (M0). Among these models, Model M1 was the model test result obtained after removing the gender attribute, and Model M2–Model M5 were the model test results obtained by experienced users with different value ranges. In the basic model M0, this study selected 25% of the newly registered users as inexperienced users and the remaining 75% as experienced users. To test the robustness of the model, we selected the top 70%, 73%, 77%, and 80% of the users with registration times from high to low as the experienced users. By examining the number of different attributes and different values of the same attribute, the influence of changes in these variables on the user interaction network was tested.

Table 4 shows the results of the model robustness test. Model M1 showed that the test results of the model were basically the same before and after removing the gender attribute. In addition, Models M2–M5 showed that after adjusting the value range of experienced users, the test results of the model were basically consistent with the basic model M0, and the parameter estimation results only had small fluctuations. The signs of the parameter estimate of gender in Models M4 and M5 were opposite to the results in Models M0, M2, and M3. The parameter estimates of emotional catharsis topics in Models M2, M3, and M4 were the same as those in Model M0. The results in M1 and M5 were opposite. The reason for these results could have been the change in the threshold range of experienced users, resulting in some newly added or removed users who preferred to communicate with the same gender, as well as a preference for discussions with emotional catharsis users. These results were consistent with the saliency in the basic model M0. Therefore, we determined that this change had little effect on the network structure. In short, by comparing the test results of Models M1–M5 with those of Model M0, we found that the basic model of this study had good robustness.

## 6. Conclusions

Based on the users’ data of the Anxiety Disorder Post Bar, with the help of the exponential random graph model, we examined the formation mechanism of the user interaction networks in ADOCs. We analyzed the effects of the network structure, user attributes, topics, and emotional intensity on the formation of the user interaction network. This research found that there was significant reciprocity in user interaction networks in ADOCs. In terms of user attributes, gender homogeneity had no obvious influence on the formation of user interaction networks in ADOCs. Experienced users in the community had obvious advantages, and experienced users were more likely to obtain replies from other members. In terms of topics, some topics showed obvious homogeneity. For example, users who preferred pathology popularization were more inclined to interact, while other topics showed obvious heterogeneity. For example, users who preferred the topic of symptoms of generalized anxiety disorder were more inclined to participate in discussions on different topics. In terms of emotional effects, users with polarized emotions were more likely to receive replies from users with positive emotions in ADOCs. The probability of interaction between two users with negative emotions was small. Users with opposite emotional polarity tended to interact, especially when the interaction was initiated by users with positive emotions to users with negative emotions.

This research had theoretical and practical significance. Based on the ERGM, combined with priority connection theory, emotion communication theory, social capital theory, as well as other theories, this paper constructed a model of an ADOC’s user interaction network. The study enriched the related research of the exponential random graph model and expanded its application scope and scenarios. In addition, the research in this study provides much needed data and perspective on interactive networks in ADOCs and revealed the formation mechanism of user interactive networks. Existing studies on social networks in online communities have mostly focused on the overall structure of the network. There have been few studies on the formation mechanism of the network structure. In addition, in previous studies, few researchers have considered the topic and emotional impacts on user interaction networks. This research enriched the understanding of the network effects of social networks.

In terms of practical significance, this research revealed how the formation of user interaction networks could be impacted by the network structure, user attributes, topics, and emotional intensity. The internal connections between users’ posts, comments, and replies and users’ personal attributes, topic preference, and emotional tendency were clarified. For example, concerning the user attribute of user registration time, experienced users who had registered for a longer time were more likely to receive replies from other users, resulting in the formation of an interactive network structure in which other users point to experienced users. Regarding the specific practical significance, the research could help community workers consider corresponding measures to promote communication between community users and increase user activity by, for example, establishing different topic sections and different topic labels. It could help users accurately locate their interests and needs and promote exchanges and interactions between users on various topics. Second, the resource advantages of experienced users in the community could be used to encourage experienced users to actively participate in topic discussions in the community. The community could also encourage experienced users to provide social support to inexperienced users through comments, replies, and so on. This could not only improve the participation of inexperienced users but also promote the development of the community and the wellbeing of community users.

Although this research contributes to the field, it also has certain limitations. This article only studied the user interaction network of the ADOC and did not consider the influence of the friend network on the user interaction network. In follow-up research, friend networks should be included to study the influence of the interaction between different networks on the network structure. In addition, the research in this article only selected static data for a period of time. In future research, we should consider the dynamic changes of these effects on the mechanism of user interaction networks.

## Figures and Tables

**Table 1:** Node attributes.

Node Attributes	Hypothesis	Variable Type	Measuring Method
Gender	H2	Binary categorical variable	1—male 0—female
Topic type	H3	Categorical variables	1—pathology popularization 2—symptoms of generalized anxiety disorder 3—panic attack records 4—emotional catharsis 5—drug efficacy 6—diagnosis and examination of hypochondriasis 7—social support
User registration time	H4	Binary categorical variable	1—experienced users 0—inexperienced users
Emotional polarity	H5	Categorical variables	2—positive emotion 1—negative emotion 0—neutral emotion

**Table 2:** Research hypothesis and network structure diagram.

Hypothesis	Factor	Diagram		
H1: Users tend to have reciprocal interactions in ADOCs.	Reciprocity	User a	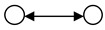	User b
H2: Users of the same gender are more likely to interact in ADOCs.	Gender	User a	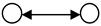 (same gender)	User b
H3: Users who prefer the same topic are more likely to interact in ADOCs.	Topic type	User a	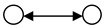 (same topic type)	User b
H4: Experienced users are more likely to receive replies from other users in ADOCs.	User registration time		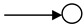	Experienced user
H5: Users with polarized emotions are more likely to receive replies from users with positive emotions in ADOCs.	Emotional polarity	User a (positive emotions)	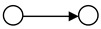	User b (polarized emotions)

**Table 3:** The exponential stochastic graph model test results.

Hypothesis	Parameter	Parameter Estimates	S.D.	*p* Value	Result
H1	reciprocity	2.898	0.297	0.000 ***	Supported
H2	gender	−0.005	0.023	0.829	Not supported
H3	topic_type_PP	0.308	0.101	0.002 **	Partially supported
	topic_type_SGAD	−0.295	0.077	0.000 ***	
	topic_type_PAR	−0.084	0.073	0.251	
	topic_type_EC	−0.021	0.059	0.726	
	topic_type_DE	0.098	0.059	0.096	
	topic_type_DEH	0.094	0.098	0.337	
	topic_type_SS	0.073	0.066	0.269	
H4	user_registration_time	0.103	0.025	0.000 ***	Supported
H5	positive_positive	0.413	0.094	0.000 ***	Supported
	negative_negative	−0.487	0.127	0.000 ***	
	positive_negative	0.534	0.090	0.000 ***	
	negative_positive	−1.191	0.142	0.000 ***	

Notes: ** *p* < 0.01; *** *p* < 0.001.

**Table 4:** Model robustness test results.

Hypothesis	Parameter	M0	M1	M2	M3	M4	M5
		Basic Model	Remove Gender	Experienced User 70%	Experienced User 73%	Experienced User 77%	Experienced User 80%
H1	reciprocity	2.898 ***	3.174 ***	3.326 ***	3.202 ***	3.711 ***	3.389 ***
H2	gender	−0.005		−0.013	−0.021	0.003	0.005
H3	topic_type_PP	0.308 ***	0.364 ***	0.400 ***	0.347 **	0.329 *	0.450 ***
	topic_type_SGAD	−0.295 ***	−0.336 ***	−0.350 ***	−0.358 ***	−0.319 ***	−0.335 ***
	topic_type_PAR	−0.084	−0.093	−0.054	−0.150	−0.090	−0.051
	topic_type_EC	−0.021	−0.031	0.003	0.033	0.014	−0.046
	topic_type_DE	0.098	0.067	0.047	0.100	0.091	0.067
	topic_type_DEH	0.094	0.143	0.124	0.109	0.209	0.162
	topic_type_SS	0.073	0.107	0.105	0.084	0.058	0.108
H4	user_registration_time	0.103 ***	0.123 ***	0.011 ***	0.126 ***	0.188 ***	0.155 ***
H5	positive_positive	0.413 ***	0.333 ***	0.403 ***	0.384 ***	0.371 ***	0.449 ***
	negative_negative	−0.487 ***	−0.488 ***	−0.483 ***	−0.594 ***	−0.585 ***	−0.493 ***
	positive_negative	0.534 ***	0.523 ***	0.494 ***	0.515 ***	0.562 ***	0.585 ***
	negative_positive	−1.191 ***	−1.168 ***	−1.232 ***	−1.246 ***	−1.149 ***	−1.083 ***

Notes: * *p* < 0.05; ** *p* < 0.01; *** *p* < 0.001.

## Data Availability

Not applicable.

## References

[1] Liu J., Zhang W., Jiang X., Zhou Y. (2020). Data Mining of the Reviews from Online Private Doctors. Telemed. e-Health.

[2] Luo A., Xin Z., Yuan Y., Wen T., Xie W., Zhong Z., Peng X., Ouyang W., Hu C., Liu F. (2020). Multidimensional Feature Classification of the Health Information Needs of Patients with Hypertension in an Online Health Community Through Analysis of 1000 Patient Question Records: Observational Study. J. Med. Internet Res..

[3] Larkings J.S., Brown P. (2018). Do biogenetic causal beliefs reduce mental illness stigma in people with mental illness and in mental health professionals? A systematic review. Int. J. Ment. Health Nurs..

[4] Rodrigues R.G., das Dores R.M., Camilo-Junior C.G., Rosa T.C. (2016). SentiHealth-Cancer: A sentiment analysis tool to help detecting mood of patients in online social networks. Int. J. Med. Inform..

[5] Deng W.H., Lv P., Yi M. (2022). How online health discussions make people perceive benefits?. Libr. Inf. Sci. Res..

[6] Guo S., Guo X., Fang Y., Vogel D. (2017). How Doctors Gain Social and Economic Returns in Online Health-Care Communities: A Professional Capital Perspective. J. Manag. Inf. Syst..

[7] Li Z., Xu X. (2020). Analysis of Network Structure and Doctor Behaviors in E-Health Communities from a Social-Capital Perspective. Int. J. Environ. Res. Public Health.

[8] Atanasova S., Kamin T., Petrič G. (2018). The benefits and challenges of online professional-patient interaction: Comparing views between users and health professional moderators in an online health community. Comput. Hum. Behav..

[9] Yan Z., Wang T., Chen Y., Zhang H. (2016). Knowledge sharing in online health communities: A social exchange theory perspective. Inf. Manag..

[10] Zhang X., Liu S., Deng Z., Chen X. (2017). Knowledge sharing motivations in online health communities: A comparative study of health professionals and normal users. Comput. Hum. Behav..

[11] Kisekka V., Giboney J.S. (2018). The Effectiveness of Health Care Information Technologies: Evaluation of Trust, Security Beliefs, and Privacy as Determinants of Health Care Outcomes. J. Med. Internet Res..

[12] Lederman R., Fan H., Smith S., Chang S. (2014). Who can you trust? Credibility assessment in online health forums. Health Policy Technol..

[13] Shiau W.-L., Dwivedi Y.K., Yang H.S. (2017). Co-citation and cluster analyses of extant literature on social networks. Int. J. Inf. Manag..

[14] Sun Y. (2020). How conversational ties are formed in an online community: A social network analysis of a tweet chat group. Inf. Commun. Soc..

[15] Said A., Bowman T.D., Abbasi R.A., Aljohani N.R., Hassan S.-U., Nawaz R. (2019). Mining network-level properties of Twitter altmetrics data. Scientometrics.

[16] Saqr M., Fors U., Tedre M. (2018). How the study of online collaborative learning can guide teachers and predict students’ performance in a medical course. BMC Med. Educ..

[17] Xu S., Zhou A. (2020). Hashtag homophily in twitter network: Examining a controversial cause-related marketing campaign. Comput. Hum. Behav..

[18] Falk A., Fischbacher U. (2006). A theory of reciprocity. Games Econ. Behav..

[19] Cropanzano R., Mitchell M.S. (2005). Social Exchange Theory: An Interdisciplinary Review. J. Manag..

[20] Zhang X., Liu S. (2021). Understanding relationship commitment and continuous knowledge sharing in online health communities: A social exchange perspective. J. Knowl. Manag..

[21] Huang J.-W., Li Y.-H. (2017). Green Innovation and Performance: The View of Organizational Capability and Social Reciprocity. J. Bus. Ethic.

[22] Ulibarri N., Scott T.A. (2017). Linking Network Structure to Collaborative Governance. J. Public Adm. Res. Theory.

[23] Harvey J., Smith A., Goulding J., Illodo I.B. (2020). Food sharing, redistribution, and waste reduction via mobile applications: A social network analysis. Ind. Mark. Manag..

[24] Robins G., Pattison P., Wang P. (2009). Closure, connectivity and degree distributions: Exponential random graph (p*) models for directed social networks. Soc. Netw..

[25] Liu X., Jiang S., Sun M., Chi X. (2020). Examining Patterns of Information Exchange and Social Support in a Web-Based Health Community: Exponential Random Graph Models. J. Med. Internet Res..

[26] McPherson M., Smith-Lovin L., Cook J.M. (2001). Birds of a Feather: Homophily in Social Networks. Annu. Rev. Sociol..

[27] Song X., Jiang S., Yan X., Chen H., Zheng X., Zeng D., Chen H., Zhang Y., Xing C., Neill D.B. (2014). Collaborative friendship networks in online healthcare communities: An exponential random graph model analysis. Smart Health.

[28] Wimmer A., Lewis K. (2010). Beyond and Below Racial Homophily: ERG Models of a Friendship Network Documented on Facebook. Am. J. Sociol..

[29] Lee S.K., Kim H., Piercy C.W. (2019). The Role of Status Differentials and Homophily in the Formation of Social Support Networks of a Voluntary Organization. Commun. Res..

[30] Johnson S.L., Faraj S., Kudaravalli S. (2014). Emergence of Power Laws in Online Communities: The Role of Social Mechanisms and Preferential Attachment. MIS Q..

[31] Bharati P., Chaudhury A. (2019). Assimilation of Big Data Innovation: Investigating the Roles of IT, Social Media, and Relational Capital. Inf. Syst. Front..

[32] Burt R.S., Staw B.M., Sutton R.I. (2000). The network structure of social capital. Research in Organizational Behavior: An Annual Series of Analytical Essays and Critical Review.

[33] Xiong J., Feng X., Tang Z. (2020). Understanding user-to-User interaction on government microblogs: An exponential random graph model with the homophily and emotional effect. Inf. Process. Manag..

[34] Goette L., Huffman D., Meier S. (2012). The Impact of Social Ties on Group Interactions: Evidence from Minimal Groups and Randomly Assigned Real Groups. Am. Econ. J. Microecon..

[35] Brady W.J., Wills J.A., Jost J.T., Tucker J.A., van Bavel J.J. (2017). Emotion shapes the diffusion of moralized content in social networks. Proc. Natl. Acad. Sci. USA.

[36] Fan R., Xu K., Zhao J. (2018). An agent-based model for emotion contagion and competition in online social media. Phys. A Stat. Mech. Appl..

[37] Robins G., Pattison P., Kalish Y., Lusher D. (2007). An introduction to exponential random graph (p*) models for social networks. Soc. Netw..

[38] Monge P.R., Contractor N. (2003). Theories of Communication Networks.

